# Supplementary material for: Gamification as an approach to improve resilience and reduce attrition in mobile mental health interventions: A randomized controlled trial
Source: PLoS One. 2020 Sep 2;15(9):e0237220. doi: 10.1371/journal.pone.0237220 (PMC7467300; doi:10.1371/journal.pone.0237220)
Supplement: S1 Protocol — (DOCX) [file pone.0237220.s002.docx]

Reference number:

Archiving endorsement:

(to be filled in by the office of the Ethics Committee)

Date: _14.10.2018

**APPLICATION**

**for the assessment of ethical questions of a research project in humans**

**to the office of the Ethics Committee of the Faculty 11**

**1. general information**

- 1. Title of the research project

First resubmission: 7_2018_Litvin_c

How Gamified mHealth Interventions Raise Life Satisfaction and Could Be the Answer To Lowering Attrition Rates.

- 1. Responsible project manager and, if applicable, deputy (name, address, telephone, e-mail address)

Prof. Dr. Markus Maier, *18.11.1971, nationality: German,

Professor of Psychology

Ludwig-Maximilians-University Munich,

Department of Psychology,

General Psychology II

Leopoldstr. 13  80802 Munich, Germany

Tel. 089 2180-5215, Fax 089 2180-3000

E-mail: Markus.Maier@psy.lmu.de

1. 3Dapplication for third-party funds no third-party donors no

1.4 Has an application with the same content already been submitted to another ethics committee?

no

If yes, please attach the vote of this ethics committee.

**Hints:**

- The Ethics Committee only assesses studies that have *not yet* been carried out, as any changes must still be feasible.

- Please add your entries to the application form in black font.

- If the application is a resubmission to the Ethics Committee of Fac. 11, the applicant must continue the application number with consecutive letters. Example: First submission: 10_2015_Mustermann_a, first re-submission: 10_2015_Mustermann_b. This application number must then also be included in the covering letter (see point 6).

The Ethics Committee also requests that the amended documents, including correspondence, be sent in electronic form with the amended text passages marked when they are resubmitted. In addition, a cover letter must be attached listing a) the comments of the Ethics Committee and b) the related changes. **If this cover letter is missing, the resubmission will not be processed.**

**2. Information on the framework conditions of the research project**

- 1. Brief information on the objectives and procedures of the research project

Since the number of mental illnesses is constantly increasing1 without sufficient therapists being available, there is an urgent need to develop intervention options that are scientifically sound, cost-effective and easily accessible to all. Since almost everyone in Europe now owns a smartphone and uses it regularly and often, it seems appropriate to offer therapy on a mobile platform. However, many of these therapies suffer from high termination rates2. Gamification could be the solution.

The aim of the study is to check whether playing a mHealth game for 6 weeks significantly increases happiness. All participants are assigned to the test or control group using a random generator. The test group gets a link to download the app. Every two weeks they are asked by e-mail to answer the questions of the Ryff PWB Scales3, Happiness Scale4, Personal Growth Scale5 and an Anxiety Likert Scale6.

The control group gets a link to a non-psychological app called 'My Story'. Every two weeks they are asked by e-mail to answer the questions of the Ryff PWB Scales, Happiness Scale, Personal Growth Scale and an Anxiety Likert Scale.

We will then compare the data and our hypothesis is that the life satisfaction values in the test group have increased significantly and the abort rate is significantly lower on average compared to other mHealth programs.

- 1. How are the study participants recruited? Also information on the study sample: description of the sample; target sample size with justification: For example, sample size planning on the basis of an a priori power analysis, or on the basis of methods for determining the desired precision of parameter estimation, for example by confidence intervals (see APA manual, 6th edition, p. 30f.). If it is unlikely that scientifically meaningful results will emerge from the study (e.g. study has too little power; results may be uninterpretable; acquisition of volunteers is problematic etc.), the effort for study participants is not justified from ethical aspects. Therefore, a scientifically justifiable justification of the sample size shall be provided in each case.

Our participants will consist of German and English speaking smartphone owners who are 18 years and older. An a priori power analysis with G* software calculated that we need a minimum of about 328 subjects to discover a significant effect value f = .1 with 80% power. Participants will be acquired through Facebook, Instagram, Twitter, MTurk and Norstat ads.

|  |
| --- |

1A. H. Weinberger, M. Gbedemah, A. M. Martinez, D. Nash, S. Galea, R. D. Goodwin. Trends in depression prevalence in the USA from 2005 to 2015: widening disparities in vulnerable groups. Psychological Medicine, 2017; 1 DOI: 10.1017/S0033291717002781

2Heleen Riper, PhD,corresponding, PhD Helen Christensen, PhD, Pim Cuijpers, PhD, Alfred Lange, PhD, Gunther Eysenbach, (2010 ). Theme Issue on E-Mental Health: A Growing Field in Internet Research. Journal of Medical Internet research. PMCID: PMC3057318

3 Ryff, C., & Keyes, C. (1995). The structure of psychological well-being revisited. Journal of Personality and Social Psychology, 69, 719–727.

4 Lyubomirsky, S., & Lepper, H. (1999). A measure of subjective happiness: Preliminary reliability and construct validation. Social Indicators Research, 46, 137-155.

5 Robitschek, C. (1999). Further validation of the Personal Growth Initiative Scale. Measurement and Evaluation in Counseling and Development, 31, 197-210.

6 Heather M. Davey, Alexandra L. Barratt, Phyllis N. Butow, Jonathan J. Deeks (2007). A one-item question with a Likert or Visual Analog Scale adequately measured current anxiety. Journal of Clinical Epidemiology, Volume 60, Issue 4, April 2007, Pages 356-360

- 1. Will participation be remunerated or will participants be promised other benefits?

A small amount of 10 € will be offered to each participant from both groups.

- 1. Is voluntary participation ensured?

Yes.

- 1. Characterization of the sample of test persons

All participants will be 18 years and older, and have a smartphone, as in the ad text is explicitly requested

- 1. Are the study participants physically stressed (e.g. by taking blood, saliva, medication or placebo, by invasive or non-invasive measurements)?

No.

- 1. Are the study participants mentally particularly stressed (e.g. by duration of activity, aversive stimuli, negative experiences)?

No, quite the opposite. This app is a game and is described as a pleasant experience by our beta testers.

- 1. Do the study participants reveal personal experiences or attitudes?

Yes, the test participants answer questions of the Ryff PWB Scales, Happiness Scale, Personal Growth Scale and an Anxiety Likert Scale.

- 1. Are study participants intentionally instructed incompletely or incorrectly about study objectives or procedures (e.g. through manipulated feedback or respondent performance)?

No.

1. **Information to be provided to the study participants prior to the examination**

*An information text for the study participants must always be submitted to the Ethics Committee; if legal representatives (e.g. parents) must also agree, a further text for them.*

**declaration of consent**

**on the scientific use of personal data**

**Studie: How Gamified mHealth Interventions Raise Life Satisfaction and Could Be the Answer to Lowering Attrition Rates.**

Dear participants,

We hereby request your consent to the scientific use of your personal data, as explained in more detail in the following information for test persons:

Within the framework of a psychological study we are looking for participants.

The study aims to determine whether playing a psychological learning game on a smartphone can help increase life satisfaction and make it easier for users to stick to a psychological intervention. If you would like to participate, you would receive a link to a mobile psychological learning game A. developed by the psychologist and PhD student Silja Litvin as part of her dissertation at the Ludwig-Maximilians-University in Munich, or a link to a non-psychological game B. In the learning game you can playfully learn psychological strategies that could eventually lead to increased life satisfaction. The study runs over a period of 6 weeks and you would complete 4 short clinical questionnaires at the beginning of the 6 weeks as well as in the middle (after 3 weeks) and at the end of the 6 weeks (presented in more detail in the study), which will help to determine whether your life satisfaction has increased with the strategies learned in the app. You will need between 5 - 10 minutes to complete these questionnaires. When downloading the apps, you will be asked to allow notifications. Please allow this during the 6 weeks, they are part of the study and serve as a weekly reminder of regular participation. In the app you will be asked to play/edit the different levels of the game. The more you get involved with the app, the better for the exam.

At the end of the trial period you will get access to App A if you only had App B and a more detailed explanation of the study, as well as the results.

Silja Litvin, the research director, is a psychologist and doctoral student at the LMU Munich. She is available at any time for questions, feedback or clarification. You can reach them privately at:

**Address of the institution:**

Ludwig-Maximilians-University of Munich

Department of Psychology

General Psychology II

Prof. Markus Maier

Leopoldstrasse 13

80802 Munich, Germany

**Contact person:**

Silja Litvin, research director of this study, psychologist and doctoral student

Royal Street 1

W1H 7LJ London

UK

E-mail: silja.litvin@campus.lmu.de

Phone: +44 7442738394

**A. General information**

I have been informed in writing by the investigator about the purpose, procedure and significance of the study as well as the benefits and risks that may be associated with it.

I have read the written information for the test persons. All my questions have been answered to my satisfaction.

I have access to a digital copy of the respondent information and consent form. I had enough time to reconsider my decision to participate in the study and to make my own decision.

My following explanations only reach as far as this was explained to me in more detail in the context of the written test person information or in the oral explanation.

My following declarations entitle and bind the above-mentioned institution.

**B. Consent to the collection of personal and clinical data**

**B 1** I agree with the collection of my data regarding my life satisfaction and hereby leave this data to the above institution.

I agree that the data collected will be used in encrypted form (i.e. in such a way that an allocation to my person is only possible via further aids - such as a reference list - under the responsibility of the institution mentioned above) and for studies with the above-mentioned question.

I am already demanding that my personal data be destroyed after the completion of these studies.

**B 2 Information on study results**

I agree that I will not provide any individual feedback about the results of the

of the study and agree to a general clarification of the study design after completion of the study.

**B 3 Free of charge**

I am aware that I will ..........receive an expense allowance for providing my data.

I am aware that I am not entitled to any compensation, royalty, or other share in any financial benefits or gains that may be obtained from research using my data.

**B 4 Information on data processing and storage**

I agree that the investigator will manually encrypt the data collected with LimeSurvey (by entering it in 2 independent and password-protected Excel tables: one with the name and an associated identification key, and another with the same identification key and the associated data). The two password-protected Excel tables are stored on the head of study's computer, which is protected by an 8-digit "Rijndael 256-bit" encrypted password. After each update, the tables are copied to a USB stick and stored as a backup in a drawer to which only the test leader has the key. The USB stick is password protected and can only be read with an appropriate password.

**B 5 Duration of storage**

I agree that the samples will be anonymized after evaluation, i.e. the encryption key will be destroyed and the data will be kept for 5 years on the password protected computer of the investigator.

**B 6 Withdrawal of consent to the use of samples**

I know that I can revoke my consent to the use of my data at any time and without giving reasons to the institution or person mentioned in the introduction and that this has no influence on my possible further medical or psychological treatment.

In the event of revocation, I agree that my data will continue to be stored for control purposes. However, I have the right to demand their deletion, provided that legal provisions do not conflict with the deletion.

I am aware that if my data is stored anonymously, it cannot be deleted at my request.

**C. Declaration of consent under data protection law**

I agree that data concerning my person will be stored and processed in encrypted form for the study with the above-mentioned question under the responsibility of the above-mentioned institution:

The data important for the clinical trial are additionally stored, transmitted and evaluated electronically in a separate case report documentation in pseudonymised form and the results are published in anonymised form.

Pseudonymisation is the processing of personal data in such a way that it can no longer be attributed to the data subjects without additional information. This additional information shall be kept separately by the investigator during this clinical trial and shall be subject to technical and organisational measures. (Your data will be collected on LimeSurvey and are subject to data protection Art. 6 para. 1 Page. 1 lit. f DSGVO. ) which ensure that the personal data cannot be unauthorisedly assigned to an identified or identifiable natural person. The data concerning you are protected against unauthorised access. The investigator creates a decryption list, which she keeps separate from the study data and the characteristics that directly identify you (surname, first name, address, date of birth, etc.). Only with the help of this list can the pseudonymised data concerning your person, as far as permitted and necessary, be reassigned to you. Decryption only takes place under the conditions described by law. The results of the above study will be published in the form of anonymised and summarised data, so that your identity will remain confidential even in this case.

**C 1 Procedure for dealing with errors**

I have understood that a step-by-step checklist will guide the investigator through each handling of the data and this will be dated for verification. If the computer of the experimenter is lost, the device will be remotely extinguished after 24 hours. If the USB stick is lost, the AES 256-bit Hardware Encryption (XTS Mode) FIPS 140-2 Level 2 Certification USB is also remotely erased within 24 hours.

**C 2 Revocation of consent to the use of data**

I know that I can revoke my consent to the use of my data at any time and without giving reasons to the institution or person mentioned in the introduction and that this has no influence on my possible further medical or psychological treatment.

In the event of revocation, I agree that my data will continue to be stored for control purposes. However, I have the right to demand their deletion, provided that legal provisions do not conflict with the deletion.

I am aware that if my data is stored anonymously, it cannot be deleted at my request.

I have read, understood and agree to all of this

Will be informed in detail about the objectives and procedures of the investigation, as well as

1. about the duration of the investigation,

Yeah, six weeks.

1. on burdens and risks arising from specific investigation procedures,

There should be no charges or risks, but we have provided our email address in case of need.

1. on remunerations and other commitments to study participants,

Yes, in the ads.

1. about the possibility of withdrawing from the participation at any time and without consequences?

Yes.

1. **Data protection**

4.1 What *personal* data is collected? (Note: According to the Federal Data Protection Act, personal data is individual information about personal or factual circumstances of a specific or identifiable natural person. Examples: name, date of birth, address, telephone number, e-mail address, genetic and medical data)

email

year of birth

sex

nationality

Ryff PWB Scales

happy scale

Personal Growth Scale

Anxiety Likert Scale

4.2 Are video or audio recordings or other behavioural registrations planned?

No.

4.3How is the anonymization or pseudonymization of the data collected secured?

Through an encryption process, see section 3.

4.4a When will the stored data be deleted?

*Note for applicants*: Personal data (e.g. name, e-mail address, place of residence, other personal data) must be deleted as soon as they are no longer required for the recruitment of test persons or for enquiries. It is advisable to include a corresponding section in the data protection declaration and declaration of consent. For example: "Your personal data will be deleted in accordance with the principles of research on humans of the Deutsche Forschungsgesellschaft (DFG). Personal data are deleted as soon as they are no longer needed for the recruitment of test persons or for enquiries".

Note: The deletion of personal data must be recorded and verified upon request.

In contrast, **completely anonymised** raw data need *not be* deleted and should be transferred to open-access scientific repositories/databases in accordance with the DFG's "Guidelines for the Handling of Research Data". Only then can the results be replicated for further research. See http://www.dfg.de/download/pdf/foerderung/antragstellung/forschungsdate[n/richtlinien_forschungsdaten.pdf](http://www.dfg.de/download/pdf/foerderung/antragstellung/forschungsdaten/richtlinien_forschungsdaten.pdf), points 2 and 3. However, the volunteers should be informed about the possible publication of the completely anonymised data in the volunteer information. See the "Example of a test person information *on data protection* at Open Data" under "8th Appendix".

In the case of **pseudonymised** data, we recommend deleting the person allocation lists after 10 years, but not the actual primary data. The primary data can then be transferred to publicly accessible repositories/databases. To distinguish *raw data* from *primary data*: Raw data are the origin recordings, e.g. answer markings in a questionnaire, drawings or audio or video recordings. *Primary data* means the first transfer of raw data into a digital format, e.g. the code "1" for a yes-answer, etc.

Primary data are therefore completely unprocessed (i.e. untransformed, non-aggregated, etc.) quantitative and qualitative data, for example

- for experiments, all manipulated and measured variables for each experimental run of each person;
- for questionnaires, the answers of each person on each item;
- in the case of free text entries, the original wording (taking into account data protection);
- in the case of video recordings or observations, the coding of the observed behaviour.

List of recommendations for open access scientific repositories/databases:

- Open Science Framework: <https://osf.io/>
- Datory of GESIS (**Dryad Digital Repository):**<http://datadryad.org/>
- PsychData (ZPID): <http://psychdata.zpid.de/>

The primary data will be deleted as soon as the experiment is finished. The processed and completely anonymised data (without e-mail addresses) will be transferred to open-access scientific repositories/databases in accordance with the DFG's "Guidelines for the Handling of Research Data".

4.4b The request must state how the pseudonymisation or anonymisation, as well as the type and time of deletion of personal data by responsible persons (which?) is to be carried out.

The nature and time of the deletion has been communicated.

4.5 Can study participants request the deletion of their data at any time?

Yes.

1. **Information on the declaration of willingness to participate in the study**

*A statement by which study participants (or their legal representatives) declare their willingness to participate in the study must be submitted to the Ethics Committee.*

**It is important to note** that the information and consent form must **not be** written separately, but as a **single** document for the information and consent form.

- 1. Does the declaration of readiness clearly refer to the participant information?

Yes.

- 1. Does it list the envisaged data protection measures?

Yes.

- 1. Does it confirm the voluntary nature of its participation in the investigation?

We don't point this out, but since it's not related to a work or a note, it should be self-explanatory.

- 1. Does it mention the right (explained under 3d) to revoke the declaration of readiness at any time?

Yes.

- 1. A contact person must be indicated for questions of the test persons regarding the study.

Yes, I have entered my name and e-mail address.

- 1. In the case of completely anonymous data, it must be indicated in the respondent information that the raw data will be made publicly accessible if they are transferred to publicly accessible scientific repositories/databases. See the example in the appendix of this document.

Yes.

1. **Note on the scope of applications submitted**

The Ethics Committee asks for concise and generally understandable proposals from the Ethics Committee. For example, it is not advisable to include a complete application to a third-party donor in the Ethics Committee application. The application itself should not normally consist of more than 5 pages plus annex. Otherwise, it is possible to postpone the processing of the application to the following session, as it is not possible to review and process the application within the 14-day period.

**7. contact**

Please address requests for comments from the Ethics Committee:

**Dean of Research Prof. Dr. Moritz Heene**

**Faculty of Psychology and Pedagogy, LMU Munich**

**Leopoldstr. 13, 80802 Munich, Germany**

# Appendix 8

**Example of a test person information on the point "Data protection with Open Data**

**Use of anonymous data**

The results and primary data of this study will be published as a scientific publication. This is done in a completely anonymous form, i.e. the data cannot be attributed to the respective participants in the study. The completely anonymized data of this study are made available as "open data" in a secure, Internet-based repository called Open Science Framework (https://osf.io/). This study thus follows the recommendations of the German Research Foundation (DFG) for quality assurance in terms of verifiability and reproducibility of scientific results, as well as optimal post-use of data.

*Responsible project manager: Maximilian Mustermann*
