# Supplementary material for: Gamification as an approach to improve resilience and reduce attrition in mobile mental health interventions: A randomized controlled trial
Source: PLoS One. 2020 Sep 2;15(9):e0237220. doi: 10.1371/journal.pone.0237220 (PMC7467300; doi:10.1371/journal.pone.0237220)
Supplement: S2 Protocol — (DOCX) [file pone.0237220.s003.docx]

Geschäftszeichen: ____________________

Eingangsvermerk: __________________

(wird von der Geschäftsstelle der Ethikkommission ausgefüllt)

Datum: _14.10.2018_________________

**ANTRAG**

**zur Beurteilung ethischer Fragen eines Forschungsvorhabens am Menschen**

**zu richten an die Geschäftsstelle der Ethikkommission der Fakultät 11**

**1. Allgemeine Angaben**

- 1. Titel des Forschungsvorhabens

Erste Wiedereinreichung: 7_2018_Litvin_c

How Gamified mHealth Interventions Raise Life Satisfaction and Could Be the Answer To Lowering Attrition Rates.

- 1. Verantwortliche/r Projektleiter/in und ggf. Stellvertreter/in (Name, Anschrift, Telefon, e-mail Adresse)

Prof. Dr. Markus Maier, *18.11.1971, Nationalität: deutsch,

Professor der Psychologie

Ludwig-Maximilians-Universität München,

Department Psychologie,

Allgemeine Psychologie II

Leopoldstr. 13  80802 München

Tel. 089 2180-5215, Fax 089 2180-3000

E-Mail: Markus.Maier@psy.lmu.de

1.3 Drittmittelantrag nein Drittmittelgeber nein

1.4 Wurde schon ein Antrag gleichen Inhalts bei einer anderen Ethikkommission gestellt?

nein

Falls ja, legen Sie bitte das Votum dieser Ethikkommission bei.

**Hinweise**:

- Die Ethikkommission beurteilt nur Studien, die *noch nicht* durchgeführt wurden, da etwaige Änderungen noch umsetzbar sein müssen.

- Bitte fügen Sie zur Ihre Einträge in das Antragsformular in schwarzer Schrift ein.

- Falls es sich beim Antrag um eine Wiedereinreichung bei der Ethikkommission der Fak. 11 handelt, muss die oder der Antragstellerin/Antragssteller die Antragsnummer selbst mit fortlaufenden Buchstaben fortführen. Bsp.: Ersteinreichung: 10_2015_Mustermann_a, erste Wiedereinreichung: 10_2015_Mustermann_b. Diese Antragsnummer ist sodann auch in das Begleitschreiben (siehe Punkt 6) einzufügen.

Die Ethikkommission bittet weiterhin bei einer Wiedereinreichung um Übersendung der geänderten Dokumente einschließlich Schriftverkehr in elektronischer Form mit Markierung der geänderten Textpassagen. Zusätzlich ist ein Begleitschreiben („cover letter“) beizufügen, das a) die Anmerkungen der Ethikkommission und b) die darauf bezogenen Änderungen aufführt. **Fehlt dieses Begleitschreiben wird die Wiedereinreichung nicht bearbeitet.**

**2. Angaben zu den Rahmenbedingungen des Forschungsvorhabens**

- 1. Kurze Angaben zu Zielen und Verfahren des Forschungsvorhabens

Da die Anzahl psychischer Erkrankungen stetig steigt^1^, ohne dass genügend Therapeuten zur Verfügung stehen, ist es dringend notwendig, Interventionsmöglichkeiten zu entwickeln, die wissenschaftlich fundiert, kostengünstig und für alle leicht zugänglich sind. Da fast jeder in Europa inzwischen ein Smartphone besitzt und es regelmäßig und oft benutzt, erscheint es angemessen, Therapie auf einer mobilen Plattform anzubieten. Viele dieser Therapien leiden jedoch an hohen Abbruchraten^2^. Gamifizierung könnte hierfür die Lösung sein.

Das Ziel der Studie ist zu überprüfen, ob das 6-wöchige Spielen eines mHealth Spiels signifikant die Lebenszufriedenheit (happiness) steigert. Alle Teilnehmer werden anhand eines Zufallsgenerators der Test- oder Kontrollgruppe zugewiesen. Die Testgruppe bekommt einen Link, um die App herunterzuladen. Alle zwei Wochen werden sie durch eine E-Mail aufgefordert, die Fragen der Ryff PWB Scales^3^, Happiness Scale^4^, Personal Growth Scale^5^ und einer Anxiety Likert Scale^6^ zu beantworten.

Die Kontrollgruppe bekommt einen Link zu einer nicht-psychologischen App namens ‚My Story‘ geschickt. Alle zwei Wochen werden sie durch eine E-Mail aufgefordert, die Fragen der Ryff PWB Scales, Happiness Scale, Personal Growth Scale und einer Anxiety Likert Scale zu beantworten.

Wir werden dann die Daten vergleichen und unsere Hypothese ist es, dass sich die Lebenszufriedenheitswerte in der Testgruppe signifikant gesteigert haben, und die Abbruchsrate im Vergleich zu anderen mHealth Programme im Durchschnitt signifikant niedriger ist

- 1. Wie werden die Studienteilnehmer rekrutiert? Ebenso Angaben zur Studienstichprobe: Beschreibung der Stichprobe; angestrebte Stichprobengröße mit Begründung: Bspw. Stichprobengrößenplanung auf Basis einer A-priori-Poweranalyse, oder auf Basis von Methoden zur Bestimmung der angestrebten Präzision der Parameterschätzung, bspw. durch Konfidenzintervalle (s. hierzu APA-Manual, 6. Auflage, S. 30f.). Ist es unwahrscheinlich, dass wissenschaftlich sinnvolle Ergebnisse aus der Studie hervorgehen (z.B. Studie weist zu geringe Power auf; Ergebnisse sind ggf. uninterpretierbar; Probanden-Akquise ist problematisch etc.), ist aus ethischen Aspekten der Aufwand für Studienteilnehmer nicht gerechtfertigt. Daher ist in jedem Fall eine wissenschaftlich nachvollziehbare Begründung der Stichprobengröße anzugeben.

Unsere Teilnehmer werden sich aus deutsch- und englischsprachigen Smartphone-Besitzern zusammensetzen, die 18 Jahre und älter sind. Eine A-priori-Poweranalyse mit G* Software errechnete, dass wir eine Mindestanzahl von ca. 328 Versuchspersonen brauchen, um einen signifikanten Effektwert f = .1 mit 80% Power zu entdecken. Die Teilnehmer werden wir mittels Facebook, Instagram, Twitter, MTurk und Norstat Anzeigen akquirierten

|  |
| --- |

1A. H. Weinberger, M. Gbedemah, A. M. Martinez, D. Nash, S. Galea, R. D. Goodwin. Trends in depression prevalence in the USA from 2005 to 2015: widening disparities in vulnerable groups. Psychological Medicine, 2017; 1 DOI: 10.1017/S0033291717002781

2Heleen Riper, PhD,corresponding, PhD Helen Christensen, PhD, Pim Cuijpers, PhD, Alfred Lange, PhD, Gunther Eysenbach, (2010 ). Theme Issue on E-Mental Health: A Growing Field in Internet Research. Journal of Medical Internet research. PMCID: PMC3057318

3 Ryff, C., & Keyes, C. (1995). The structure of psychological well-being revisited. Journal of Personality and Social Psychology, 69, 719–727.

4 Lyubomirsky, S., & Lepper, H. (1999). A measure of subjective happiness: Preliminary reliability and construct validation. Social Indicators Research, 46, 137-155.

5 Robitschek, C. (1999). Further validation of the Personal Growth Initiative Scale. Measurement and Evaluation in Counseling and Development, 31, 197-210.

6 Heather M. Davey, Alexandra L. Barratt, Phyllis N. Butow, Jonathan J. Deeks (2007). A one-item question with a Likert or Visual Analog Scale adequately measured current anxiety. Journal of Clinical Epidemiology, Volume 60, Issue 4, April 2007, Pages 356-360

- 1. Wird die Teilnahme vergütet bzw. werden Teilnehmern andere Vorteile zugesagt?

Eine geringe Summe in Höhe von 10 € wird jedem Teilnehmer aus beiden Gruppen angeboten.

- 1. Ist die Freiwilligkeit der Teilnahme gesichert?

Ja.

- 1. Charakterisierung der Probandenstichprobe

Alle Teilnehmer werden 18 Jahre und älter sein, und ein Smartphone besitzen, wie im Anzeigen Text explizit angefragt wird

- 1. Werden die Studienteilnehmer körperlich beansprucht (z.B. durch Entnahme von Blut, Speichel, durch Medikamenten- oder Placebogaben, durch invasive oder nicht-invasive Messungen)?

Nein.

- 1. Werden die Studienteilnehmer mental besonders beansprucht (z.B. durch Tätigkeitsdauer, aversive Reize, negative Erfahrungen)?

Nein, ganz im Gegenteil. Die vorliegende App ist ein Spiel und wird als angenehme Erfahrung von unseren Beta-Testern beschrieben.

- 1. Geben die Studienteilnehmer persönliche Erfahrungen oder Einstellungen preis?

Ja, die Versuchsteilnehmer beantworten Fragen der Ryff PWB Scales, Happiness Scale, Personal Growth Scale und einer Anxiety Likert Scale.

- 1. Werden die Studienteilnehmer absichtlich unvollständig oder falsch über Untersuchungsziele oder Verfahren instruiert (z.B. durch manipulierter Rückmeldungen oder Probanden-Leistungen)?

Nein.

1. **Angaben über die Informierung der Studienteilnehmer vor der Untersuchung**

*Ein Informationstext für die Studienteilnehmer muss der Ethikkommission in jedem Fall vorgelegt werden; falls auch gesetzliche Vertreter (z.B. Eltern) zustimmen müssen, ein weiterer Text für diese.*

**Einwilligungserklärung**

**zur wissenschaftlichen Verwendung personenbezogener Daten**

**Studie: How Gamified mHealth Interventions Raise Life Satisfaction and Could Be the Answer to Lowering Attrition Rates.**

Sehr geehrte Teilnehmer,

hiermit bitten wir Sie um Ihr Einverständnis zur wissenschaftlichen Verwendung Ihrer personenbezogenen Daten, wie sie Ihnen in der folgenden Probandeninformation näher erläutert wird:

Im Rahmen einer psychologischen Studie suchen wir Teilnehmer.

In der Studie geht es darum festzustellen, ob das Spielen eines psychologischen Lern-Spieles auf einem Smartphone helfen kann, Lebenszufriedenheit zu erhöhen und es den Benutzern erleichtert an einer psychologischen Intervention dranzubleiben. Falls Sie teilnehmen möchten, würden Sie einen Link zu einem mobilen psychologischem Lern-Spiel A. bekommen, die von der Psychologin und Doktorandin Silja Litvin im Rahmen ihrer Dissertation an der Ludwig-Maximilians-Universität in München entwickelt wurde, oder einen Link zu einem nicht-psychologischen Spiel B. In dem Lern-Spiel können sie spielerisch psychologische Strategien lernen die eventuel dazu führen könnten die Lebenszufriedenheit zu erhöhen. Die Studie läuft über einen Zeitraum von 6 Wochen, und Sie würden am Anfang der 6 Wochen sowie in der Mitte (nach 3 Wochen) und am Ende der 6 Wochen 4 kurze klinische Fragebögen ausfüllen (in der Studie näher vorgestellt), die dabei helfen festzustellen, ob Ihre Lebenszufriedenheit mit den in der App erlernten Strategien gesteigert wurde. Sie benötigen zwischen 5 – 10 Minuten zum Ausfüllen dieser Fragebögen. Beim Herunterladen der Apps werden Sie gebeten, Notifikationen zuzulassen. Erlauben Sie dies während der 6 Wochen bitte, sie gehören zur Studie und dienen der wöchentlichen Erinnerung an die regelmäßige Teilnahme. In der App werden Sie gebeten, die verschiedenen Ebenen des Spieles zu spielen/bearbeiten. Je mehr Sie sich mit der App auseinandersetzen, desto besser für die Untersuchung.

Am Ende der Laufzeit erhalten Sie Zugang zu App A falls Sie nur App B hatten und eine tiefergehende Aufklärung über die Studie, als auch die Ergebnisse.

Die Versuchsleiterin, Silja Litvin, ist Psychologin und Doktorandin an der LMU München. Sie steht jederzeit für Fragen, Feedback oder Aufklärung zur Verfügung. Sie erreichen sie privat unter:

**Adresse der Institution:**

Ludwig-Maximilians-Universität München

Fachbereich Psychologie

Allgemeine Psychologie II

Herrn Prof. Markus Maier

Leopoldstraße 13

80802 München

**Ansprechpartner:**

Silja Litvin, Versuchsleiterin dieser Studie, Psychologin und Doktorandin

Royal Street 1

W1H 7LJ London

UK

E-Mail: silja.litvin@campus.lmu.de

Telefon: +44 7442738394

**A. Allgemeines**

Ich bin durch die Versuchsleiterin über den Zweck, den Ablauf und die Bedeutung der Studie sowie die Vorteile und Risiken, die damit verbunden sein können, schriftlich aufgeklärt worden.

Die schriftliche Probandeninformation habe ich gelesen. Alle meine Fragen sind zu meiner Zufriedenheit beantwortet worden.

Ich habe Zugang zu einer digitalen Kopie der Probandeninformation und Einverständniserklärung. Ich hatte genügend Zeit, um meine Entscheidung zur Studienteilnahme zu überdenken und frei zu treffen.

Meine folgenden Erklärungen reichen nur so weit, wie mir dies im Rahmen der schriftlichen Probandeninformation bzw. in der mündlichen Erläuterung näher dargelegt wurde.

Meine folgenden Erklärungen berechtigen und verpflichten die oben einleitend genannte Institution.

**B. Einwilligung in die Erhebung persönlicher und klinischer Daten**

**B 1** Ich bin mit der Erhebung meiner Daten bezüglich meiner Lebenszufriedenheit einverstanden und überlasse diese Daten hiermit der oben genannten Institution.

Ich stimme zu, dass die erhobenen Daten unter der Verantwortung der oben genannten Institution in verschlüsselter Form (d.h. so, dass eine Zuordnung zu meiner Person nur über weitere Hilfsmittel – etwa eine Referenzliste – möglich ist) und für Studien mit der oben genannten Fragestellung verwendet wird.

Ich verlange bereits heute, dass meine Personenbezogenen Daten nach Abschluss der genannten Studien vernichtet werden.

**B 2 Information über Studienergebnisse**

Ich bin damit einverstanden, dass ich keine individuellen Rückinformationen über die Ergebnisse

der Studie erhalte und bin mit einer allgemeinen Aufklärung über den Studienaufbau nach Beendigung der Studie einverstanden.

**B 3 Unentgeltlichkeit**

Ich bin mir bewusst, dass ich für die Überlassung meiner Daten eine Aufwandsentschädigung in Höhe von .......... erhalte.

Ich bin mir bewusst, keinerlei Ansprüche auf Vergütung, Tantieme oder sonstige Beteiligung an finanziellen Vorteilen und Gewinnen zu haben, die möglicherweise auf der Basis der Forschung mit meinen Daten erlangt werden.

**B 4 Angaben zur Datenverarbeitung und Aufbewahrung**

Ich bin damit einverstanden, dass die Versuchsleiterin die mit LimeSurvey erhobene Daten manuell verschlüsseln wird (durch das Eintragen in 2 unabhängige und Passwortgeschütze Excel Tabellen: eine mit dem Namen und einem dazugehörigen Identifikationsschlüssel, und eine andere mit dem gleichen Identifikationsschlüssel und den dazugehörigen Daten). Die zwei Passwortgeschützen Excel Tabellen werden auf dem Computer der Studienleiterin gespeichert, der mit einem 8-stelligen "Rijndael 256-Bit"-verschlüsseltem Passwort geschützt ist. Nach jedem Update werden die Tabellen auf einen USB Stick kopiert und als Sicherung in einer Schublade aufbewahrt, zu der nur die Versuchsleiterin den Schlüssel hat. Der USB Stick ist Passwortgeschützt und kann nur mit einem entsprechenden Passwort gelesen werden.

**B 5 Dauer der Aufbewahrung**

Ich bin damit einverstanden, dass die Proben nach der Auswertung anonymisiert werden, d.h. der Verschlüsselungscode wird vernichtet und die Daten werden für 5 Jahre auf dem Passwortgeschützten Computer der Versuchsleiterin aufbewahrt.

**B 6 Widerruf der Zustimmung zur Probenverwendung**

Ich weiß, dass ich meine Zustimmung zur Verwendung meiner Daten jederzeit und ohne Angabe von Gründen gegenüber der einleitend genannten Institution bzw. Person widerrufen kann, und dass dies keinen Einfluss auf meine etwaig weiteren ärztlichen oder psychologischen Behandlungen hat.

Im Falle des Widerrufs bin ich damit einverstanden, dass meine Daten zu Kontrollzwecken weiterhin gespeichert bleiben. Ich habe jedoch das Recht, deren Löschung zu verlangen, sofern gesetzliche Bestimmungen der Löschung nicht entgegenstehen.

Ich bin mir bewusst, dass im Falle einer anonymisierten Speicherung meiner Daten deren Löschung auf meinen Wunsch nicht möglich ist.

**C. Datenschutzrechtliche Einwilligungserklärung**

Ich stimme zu, dass Daten, die meine Person betreffen unter der Verantwortung der oben genannten Institution in verschlüsselter Form für die Studie mit der oben genannten Fragestellung gespeichert und verarbeitet werden:

Die für die klinische Prüfung wichtigen Daten werden zusätzlich in pseudonymisierter Form in einer gesonderten Fallberichtsdokumentation elektronisch gespeichert, übermittelt und ausgewertet und die Ergebnisse in anonymisierter Form veröffentlicht.

Pseudonymisierung ist die Verarbeitung personenbezogener Daten in einer Weise, dass sie ohne Hinzuziehung zusätzlicher Informationen den betroffenen Personen nicht mehr zugeordnet werden können. Diese zusätzlichen Informationen werden im Rahmen dieser klinischen Prüfung von der Versuchsleiterin gesondert aufbewahrt und unterliegen technischen und organisatorischen Maßnahmen. (Ihre Daten werden auf LimeSurvey erhoben und unterliegen dem Datenschutz Art. 6 para. 1 Page. 1 lit. f DSGVO.) die gewährleisten, dass die personenbezogenen Daten nicht unbefugt einer identifizierten oder identifizierbaren natürlichen Person zugewiesen werden können. Die Sie betreffenden Daten sind gegen unbefugten Zugriff gesichert. Die Versuchsleiterin erstellt eine Entschlüsselungsliste, die sie getrennt von den Studiendaten und den Sie unmittelbar identifizierenden Merkmalen (Name, Vorname, Anschrift, Geburtsdatum etc.) aufbewahrt. Nur mit Hilfe dieser Liste können die pseudonymisierten Daten zu Ihrer Person, soweit erlaubt und erforderlich, Ihnen wieder zugeordnet werden. Eine Entschlüsselung erfolgt nur unter den vom Gesetz beschrieben Voraussetzungen. Die Veröffentlichung der Ergebnisse der o.g. Studie erfolgt in Form von anonymisierten und zusammengefassten Daten, sodass Ihre Identität auch in diesem Fall vertraulich bleibt.

**C 1 Verfahren zum Umgang mit Fehlern**

Ich habe verstanden, dass eine Schritt-für-Schritt Checkliste die Versuchsleiterin durch jeden Umgang mit den Daten führen wird und dies mit Datum zur Überprüfung versehen sein wird. Bei einem etwaigen Verlust des Computers der Versuchsleiterin wird das Gerät nach 24 Stunden ferngelöscht. Beim Verlust des USB Sticks wird der AES 256-bit Hardware Encryption (XTS Mode) FIPS 140-2 Level 2 Certification USB ebenfalls innerhalb von 24 Stunden ferngelöscht.

**C 2 Widerruf der Zustimmung zur Datenverwendung**

Ich weiß, dass ich meine Zustimmung zur Verwendung meiner Daten jederzeit und ohne Angabe von Gründen gegenüber der einleitend genannten Institution bzw. Person widerrufen kann, und dass dies keinen Einfluss auf meine etwaig weiteren ärztlichen oder psychologischen Behandlungen hat.

Im Falle des Widerrufs bin ich damit einverstanden, dass meine Daten zu Kontrollzwecken weiterhin gespeichert bleiben. Ich habe jedoch das Recht, deren Löschung zu verlangen, sofern gesetzliche Bestimmungen der Löschung nicht entgegenstehen.

Ich bin mir bewusst, dass im Falle einer anonymisierten Speicherung meiner Daten deren Löschung auf meinen Wunsch nicht möglich ist.

Ich habe alles gelesen, verstanden und bin einverstanden

Wird detailliert über Ziele und Verfahren der Untersuchung aufgeklärt, wie auch

1. über die Dauer der Untersuchung,

Ja, 6 Wochen.

1. über Belastungen und Risiken durch spezifische Untersuchungsverfahren,

Es sollte keine Belastungen oder Risiken geben, aber wir haben für den Fall der Fälle unsere E-Mail-Adresse mitgeteilt.

1. über Vergütungen und andere Zusagen an die Studienteilnehmer,

Ja, in den Anzeigen.

1. über die jederzeitige und folgenlose Rücktrittsmöglichkeit von der Teilnahme-bereitschaft?

Ja.

1. **Angaben zum Datenschutz**

4.1 Welche *personenbezogenen* Daten werden erhoben? (Hinweis: Nach dem Bundesdatenschutzgesetz sind personenbezogene Daten Einzelangaben über persönliche oder sachliche Verhältnisse einer bestimmten oder bestimmbaren natürlichen Person. Beispiele: Name, Geburtsdatum, Anschrift, Telefonnummer, E-Mailadresse, genetische Daten und Krankendaten)

E-Mail

Geburtsjahr

Geschlecht

Nationalität

Ryff PWB Scales

Happiness Scale

Personal Growth Scale

Anxiety Likert Scale

4.2 Sind Video- oder Tonaufnahmen oder andere Verhaltensregistrierungen vorgesehen?

Nein.

4.3 Wie wird die Anonymisierung oder Pseudonymisierung der erhobenen Daten gesichert?

Durch einen Verschlüsselungsvorgang, sehe Abschnitt 3.

4.4a Wann werden die gespeicherten Daten gelöscht?

*Hinweis für Antragsteller*: Die personenbezogenen Daten (z.B. Erhebung von Name, Emailadresse, Wohnort, weitere personenbezogene Daten) sind, sobald sie nicht mehr zur Rekrutierung der Probanden oder für Nachfragen benötigt werden zu löschen. Es bietet sich an, einen entsprechenden Abschnitt in die Datenschutzerklärung und Einverständniserklärung einzufügen. Beispielsweise: „Die Löschung Ihrer personenbezogenen Daten erfolgt gemäß der Grundsätze der Forschung am Menschen der Deutschen Forschungsgesellschaft (DFG). Die personenbezogenen Daten werden gelöscht, sobald sie nicht mehr zur Rekrutierung der Probanden oder für Nachfragen benötigt werden“.

Beachten: Die erfolgte Löschung personenbezogener Daten ist zu protokollieren und auf Anforderung nachzuweisen.

Demgegenüber müssen **vollständig** **anonymisierte** Rohdaten *nicht* gelöscht werden und sollten gemäß der „Leitlinien zum Umgang mit Forschungsdaten“ der DFG in offen zugängliche wissenschaftliche Repositorien/Datenbanken überführt werden. Nur dann ist eine Replizierbarkeit der Ergebnisse für weiterführende Forschungen gewährleistet. Siehe hierzu <http://www.dfg.de/download/pdf/foerderung/antragstellung/forschungsdaten/richtlinien_forschungsdaten.pdf>, Punkt 2 und 3. Über die mögliche Veröffentlichung der vollständig anonymisierten Daten sollten aber die Probanden in der Probandeninformation informiert werden. Siehe hierzu das „Musterbeispiel einer Probandeninformation *zum Datenschutz* bei Open Data“ unter „8. Anhang“.

Bei **pseudonymisierten** Daten empfehlen wir nach 10 Jahren die Personen-Zuordnungslisten, aber nicht die eigentlichen Primärdaten zu löschen. Die Primärdaten können dann in öffentlich zugängliche Repositorien/Datenbanken überführt werden. Zur Unterscheidung von *Rohdaten* gegenüber *Primärdaten*: Rohdaten sind die Ursprungsaufzeichnungen, z.B. Antwortmarkierungen in einem Fragebogen, Zeichnungen oder Audio- oder Videoaufnahmen. Mit *Primärdaten* ist die erste Übertragung der Rohdaten in ein digitales Format gemeint, also z.B. der Code „1“ für eine Ja-Antwort usw.

Primärdaten sind also vollkommen unbearbeitete (d.h. untransformierte, nicht aggregierte etc.) quantitative und qualitative Daten, zum Beispiel

- bei Experimenten alle manipulierten und gemessenen Variablen für jeden Experimentaldurchgang jeder Person;
- bei Fragebögen die Antworten jeder Person auf jedem Item;
- bei Freitext-Eingaben der Originalwortlaut (unter Berücksichtigung des Datenschutzes);
- bei Videoaufnahmen bzw. Beobachtungen die Kodierung des beobachteten Verhaltens.

Liste von Empfehlungen zu offen zugänglichen wissenschaftlichen Repositorien/Datenbanken:

- Open Science Framework: <https://osf.io/>
- Datorium von GESIS (**Dryad Digital Repository):** <http://datadryad.org/>
- PsychData (ZPID): <http://psychdata.zpid.de/>

Die Primärdaten werden gelöscht, sobald der Versuch fertig ist. Die bearbeiteten und komplett anonymisierten Daten (ohne E-Mail-Adressen) werden gemäß der „Leitlinien zum Umgang mit Forschungsdaten“ der DFG in offen zugängliche wissenschaftliche Repositorien/Datenbanken überführt werden.

4.4b Im Antrag ist auszuführen wie die Pseudonymisierung oder Anonymisierung, sowie Art und Zeit der Löschung der personenbezogenen Daten durch verantwortliche Personen (welche?) durchgeführt wird.

Die Art und der Zeitpunkt der Löschung wurde mitgeteilt.

4.5 Können Studienteilnehmer jederzeit die Löschung ihrer Daten verlangen?

Ja.

1. **Angaben zur Erklärung der Bereitschaft, an der Studie teilzunehmen**

*Eine Erklärung, mit der Studienteilnehmer (oder deren gesetzliche Vertreter) die Bereitschaft zur Teilnahme an der Untersuchung bekunden, muss der Ethikkommission in jedem Fall vorgelegt werden.*

**Unbedingt zu beachten**: die Probandeninformation und Einverständniserklärung **nicht** separat verfassen, sondern als ein **einziges** Dokument zur Probandeninformationen und Einverständniserklärung.

- 1. Nimmt die Bereitschaftserklärung eindeutig Bezug auf die Teilnehmer-Information?

Ja.

- 1. Führt sie die vorgesehenen Maßnahmen zum Datenschutz auf?

Ja.

- 1. Bestätigt sie die Freiwilligkeit der Teilnahme an der Untersuchung?

Wir weisen nicht extra darauf hin, aber da es nicht im Zusammenhang mit einer Arbeit oder einer Note ist, sollte es selbsterklärend sein.

- 1. Erwähnt sie das (unter 3d erläuterte) Recht, die Bereitschaftserklärung jederzeit widerrufen zu können?

Ja.

- 1. Ein Ansprechpartner für Rückfrage der ProbandInnen zur Studie muss angegeben werden.

Ja, ich habe meinen Namen und eine E-Mail-Adresse angegeben.

- 1. Bei vollständig anonymisierten Daten muss in der Probandeninformation darauf hingewiesen werden, dass die Rohdaten öffentlich zugänglich gemacht werden, wenn sie in öffentlich zugänglich wissenschaftliche Repositorien/Datenbanken überführt werden. Siehe hierzu das Beispiel im Anhang dieses Dokuments.

Ja.

1. **Hinweis zum Umfang eingereichter Anträge**

Die Ethikkommission bittet um prägnante und allgemeinverständlich formulierte Ethikkommissionsanträge. Es ist beispielsweise davon abzusehen, einen kompletten Antrag an einen Drittmittelgeber in den Ethikkommissionsantrag einzufügen. Der Antrag selbst sollte in der Regel nicht mehr als 5 Seiten plus Anhang umfassen. Ansonsten ist Verschiebung der Antragsbearbeitung in die folgende Sitzung möglich, da eine Durchsicht und Bearbeitung nicht innerhalb der Frist von 14 Tagen möglich ist.

**7. Kontakt**

Anfragen für Stellungnahmen der Ethikkommission richten Sie bitte an:

**Forschungsdekan Prof. Dr. Moritz Heene**

**Fakultät für Psychologie und Pädagogik der LMU München**

**Leopoldstr. 13, 80802 München**

# 8. Anhang

**Musterbeispiel einer Probandeninformation zum Punkt „Datenschutz bei Open Data“**

**Verwendung der anonymisierten Daten**

Die Ergebnisse und Primärdaten dieser Studie werden als wissenschaftliche Publikation veröffentlicht. Dies geschieht in vollständig anonymisierter Form, d.h. ohne dass die Daten den jeweiligen TeilnehmerInnen an der Studie zugeordnet werden können. Die vollständig anonymisierten Daten dieser Studie werden als "open data" in einem sicheren, internetbasierten Repositorium namens Open Science Framework (https://osf.io/) zugänglich gemacht. Damit folgt diese Studie den Empfehlungen der Deutschen Forschungsgemeinschaft (DFG) zur Qualitätssicherung in Bezug auf Nachprüfbarkeit und Reproduzierbarkeit wissenschaftlicher Ergebnisse, sowie der optimalen Datennachnutzung.

*Verantwortlicher Projektleiter: Maximilian Mustermann*
